# Comparative Genomics Analysis of Repetitive Elements in Ten Gymnosperm Species: “Dark Repeatome” and Its Abundance in Conifer and *Gnetum* Species

**DOI:** 10.3390/life11111234

**Published:** 2021-11-15

**Authors:** Avi Titievsky, Yuliya A. Putintseva, Elizaveta A. Taranenko, Sofya Baskin, Natalia V. Oreshkova, Elia Brodsky, Alexandra V. Sharova, Vadim V. Sharov, Julia Panov, Dmitry A. Kuzmin, Leonid Brodsky, Konstantin V. Krutovsky

**Affiliations:** 1 Tauber Bioinformatics Research Center, University of Haifa, Haifa 3498838, Israel; atitievsk@staff.haifa.ac.il (A.T.); etaranenko@sfu-kras.ru (E.A.T.); sfbskn@gmail.com (S.B.); alade@sfu-kras.ru (A.V.S.); vsharov@sfu-kras.ru (V.V.S.); juliapanov.uni@gmail.com (J.P.); lbrodsky@research.haifa.ac.il (L.B.); 2 Laboratory of Forest Genomics, Genome Research and Education Center, Institute of Fundamental Biology and Biotechnology, Siberian Federal University, 660036 Krasnoyarsk, Russia; yputintseva@sfu-kras.ru (Y.A.P.); oreshkova@ksc.krasn.ru (N.V.O.); 3 Laboratory of Genomic Research and Biotechnology, Federal Research Center “Krasnoyarsk Science Center of the Siberian Branch of the Russian Academy of Sciences”, 660036 Krasnoyarsk, Russia; 4 Department of Genomics and Bioinformatics, Institute of Fundamental Biology and Biotechnology, Siberian Federal University, 660074 Krasnoyarsk, Russia; 5 Scientific and Methodological Center, G. F. Morozov Voronezh State University of Forestry and Technologies, 394087 Voronezh, Russia; 6 Pine Biotech Inc., New Orleans, LA 70112, USA; elia@pine.bio; 7 Department of High Performance Computing, Institute of Space and Information Technologies, Siberian Federal University, 660074 Krasnoyarsk, Russia; dkuzmin@sfu-kras.ru; 8 Department of Forest Genetics and Forest Tree Breeding, Georg-August University of Göttingen, 37077 Göttingen, Germany; 9 Center for Integrated Breeding Research, Georg-August University of Göttingen, 37075 Göttingen, Germany; 10 Laboratory of Population Genetics, N. I. Vavilov Institute of General Genetics, Russian Academy of Sciences, 119333 Moscow, Russia

**Keywords:** gymnosperms, repetitive elements, principal component analysis

## Abstract

Repetitive elements (RE) and transposons (TE) can comprise up to 80% of some plant genomes and may be essential for regulating their evolution and adaptation. The “repeatome” information is often unavailable in assembled genomes because genomic areas of repeats are challenging to assemble and are often missing from final assembly. However, raw genomic sequencing data contain rich information about RE/TEs. Here, raw genomic NGS reads of 10 gymnosperm species were studied for the content and abundance patterns of their “repeatome”. We utilized a combination of alignment on databases of repetitive elements and de novo assembly of highly repetitive sequences from genomic sequencing reads to characterize and calculate the abundance of known and putative repetitive elements in the genomes of 10 conifer plants: *Pinus taeda*, *Pinus sylvestris*, *Pinus sibirica*, *Picea glauca*, *Picea abies*, *Abies sibirica*, *Larix sibirica*, *Juniperus communis*, *Taxus baccata*, and *Gnetum gnemon*. We found that genome abundances of known and newly discovered putative repeats are specific to phylogenetically close groups of species and match biological taxa. The grouping of species based on abundances of known repeats closely matches the grouping based on abundances of newly discovered putative repeats (*kChains*) and matches the known taxonomic relations.

## 1. Introduction

Gymnosperm genomes are relatively large and variable in size, spanning from 12 Gb in *Larix sibirica*, 20 Gb in *Picea* [1,2], and up to 30 Gb in some *Pinus* species [3,4]. Almost 80% of gymnosperm genomes are constituted by repetitive elements (REs), including transposable elements (TE) [1,2,5,6,7,8,9]. The number of well-supported genes in conifer genomes is similar to *Arabidopsis thaliana*, whose genome is about 100 times smaller [2]. A combination of polyploidy, high levels of repetitive DNA (RE) amplification [10], and low rates of DNA removal due to the lack of an efficient transposon suppression mechanism [2,3] can explain these differences in genome sizes.

Only a few whole-genome sequencing data sets of conifers are currently available [1,2,5,6,7,9,11,12] compared to more than 100 other plant genomes that have been assembled [13]. The size is not the only challenge for conifer genome study and de novo assembly. Conifers have a high embryonic genetic load of about eight lethal equivalents per embryo on average [14]. In addition, inbred lines that usually facilitate genome assembly do not exist in conifers [2]. Conifers are highly heterozygous, although their nucleotide substitution rates are lower than those of most angiosperms [15] and they have high synteny [11,16]. In addition to high allelic variation [15,17], they have high non-allelic variation due to more complex and extended multiple gene families [18,19,20]. All these factors add to the complexity of in-depth genomic studies.

Repetitive elements (REs) may be among the most important internal sources of genotypic variation between species due to their ability to generate mutations, alter gene expression, and promote chromosomal aberrations [21,22,23,24,25,26]. The key environmental factors affecting plant evolution include wildfires, droughts, and frost. Such extreme stress factors can activate retrotransposons [21,22,27], leading to variation in abundance of repetitive elements in different species [28]. Previously, it has been suggested that the abundance of RE in the genome of a species carries phylogenetic signals; moreover, repetitive elements in the genomes of biologically related species are differentially amplified and evolve independently after speciation [29,30]. Thus, the genomic abundance of repetitive elements may be used for inferring the evolutionary relationship between organisms.

Here, we identified and analyzed the most abundant repetitive DNA, both known families of repeats and newly identified putative repeats, in the genomes of ten gymnosperm plants: *Pinus taeda*, *Pinus sylvestris*, *Pinus sibirica*, *Picea glauca*, *Picea abies*, *Abies sibirica*, *Larix sibirica*, *Juniperus communis*, *Taxus baccata*, and *Gnetum gnemon*. Comparison of the abundance of repetitive DNA revealed that the abundances of repeats are specific to groups of species and match biological taxa. Moreover, the abundance of repeats contains phylogenetic signal and the phylogenetic relations inferred from this signal are close to, but do not exactly match, the known phylogeny of the studied plants.

## 2. Materials and Methods

### 2.1. Whole-Genome Sequencing Data

We collected a set of whole-genome sequencing data generated from ten conifer species with three replicates per species (Table S1). To normalize the samples in size and thus be able to compare the abundance of repetitive elements in each sample, we randomly chose 10 Gb of raw genomic reads from each sample, 300 Gb of raw sequencing reads in total, for our analysis.

### 2.2. Bioinformatics Analysis

**Figure 1:**
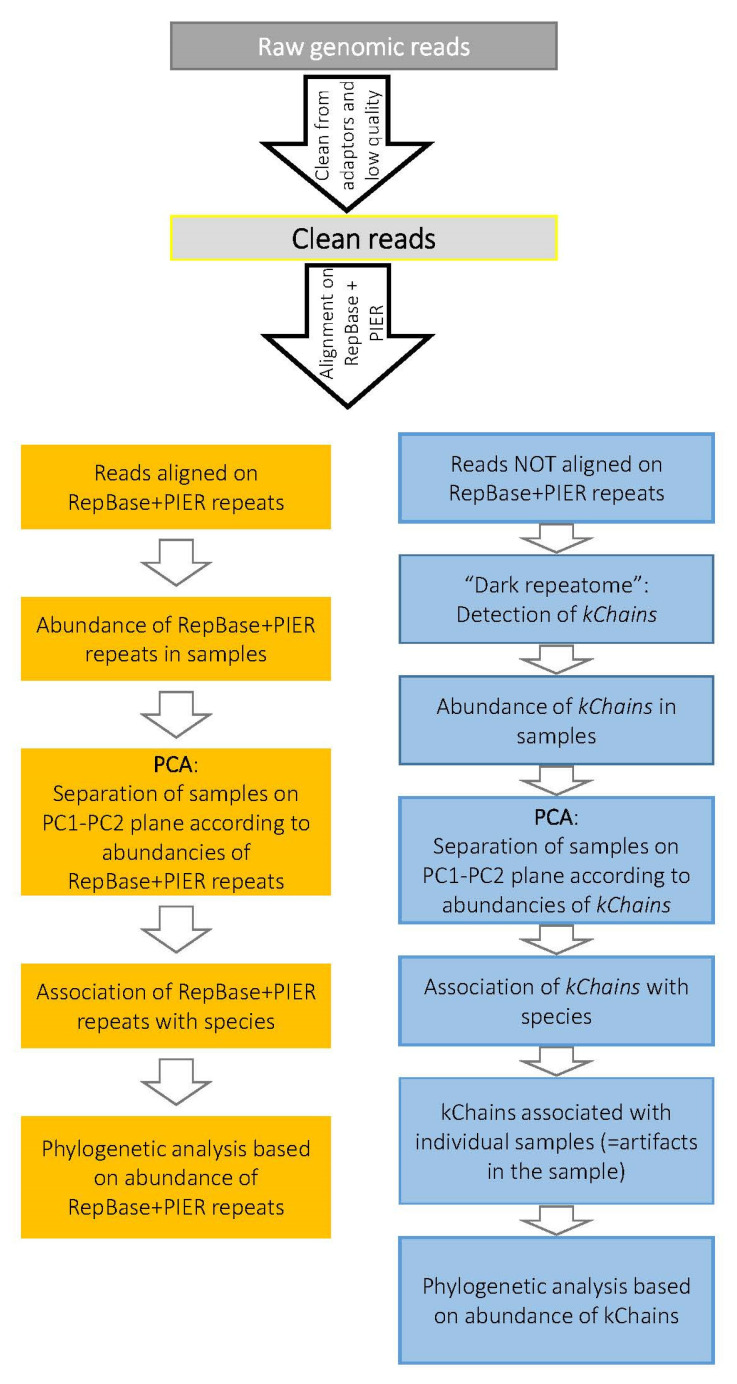
Schematic presentation of analysis of repetitive elements in conifer and *Gnetum genomes*.

The workflow for studying the repetitive DNA of ten conifer species is presented in Figure 1. Shortly, adapters were trimmed, and low-quality reads were excluded. Then, the remaining reads were aligned to RepBase and PIER databases of known repetitive elements. The abundance matrix of RepBase and PIER repeats was used in downstream analyses as the abundance of ‘known RE’. Next, using 10% of reads unaligned to RepBase and PIER, we assembled highly repetitive *kChain* sequences. Alignment of all reads to the assembled *kChains* gave an abundance matrix of *kChains* that was used in downstream analyses.

### 2.3. Read Cleaning and Quality Assurance

Prior to the analysis of RE, we performed extensive quality assurance of the raw genomic reads. The reads were trimmed from adaptors using Trimmomatic algorithm [31] with default parameters. As we collected the sequencing datasets from NCBI database, we did not have information on the adapters used for each sequencing run. Thus, we did not add any adapters to the default list used by Trimmomatic. The low-quality reads were filtered out. One of the *Pinus sibirica* samples has not been preprocessed to provide a negative control for the quality control procedure.

### 2.4. Alignment of Genomics Reads onto Repeat Sequences: RE Abundance Calculation

Alignment of the genomics reads on RepBase [7,32], the *Pinus taeda* specific repetitive element database PIER [8], and on the newly detected REs (*Kchains*) was performed with relatively relaxed thresholds. We used Bowtie2 mapper in local mode, looking only for one alignment [33]. The relaxed mapping procedure was required for two reasons: firstly, the raw sequencing reads from different plant species were aligned to the same known REs that might be slightly varied especially in different taxonomic groups. Secondly, the reads not aligned to known RE were assembled into consensus putative repeats (*kChains*). Again, alignment on *kChain* sequences must be flexible to ensure successful alignment of slightly different reads from taxonomically different plants on consensus sequences. The abundances of RE and *kChains* in each sample were estimated as the median of per-position coverage across all positions of the repeat sequence.

The raw abundance table was transformed to a natural logarithmic scale, quantile normalized, and filtered so that only RE with high abundance (in each section, the exact abundance is stated) at least in one sample remain before all downstream analyses.

### 2.5. Putative Repetitive Elements—kChains

Putative repetitive elements were extracted from the raw whole genome sequencing reads by utilizing the tBiClustering algorithm [34]. Shortly, the tBiClustering approach for finding repeats in raw reads is based on the unsupervised detection of dense associations between k-mers (k-tuples in sequences, k = 12, 15) and short sequence fragments of the genome (NGS reads).

The task can be defined as a search for densely connected subsets of vertices of two disjoint types in a bi-partite graph *G* {*V*_1_, *V*_2_, *E*}. Here, two types of vertices (*V*_1_ and *V*_2_) are k-mers and the raw genomics reads. A k-mer and a read are linked by an edge if the k-mer is a sub-sequence of the read. The tBiClustering approach exploits the co-clustering idea: find two subsets, *V*_s_ ⊂ *V*_1_ and *V*_t_ ⊂ *V*_2_, simultaneously, which are densely connected. The density of a bipartite sub-graph *G*_st_ is defined as: d*G*_st_ = |*E*_st_|/(|*V*_s_| · |*V*_t_|). According to this definition, d*G*_st_ ∈ [0, 1], and a subgraph has the density one if and only if it is a biclique. The assembled k-mers inside every tBiCluster are repetitive sub-sequences of the genomic reads (*kChains*).

We used 10% of randomly selected PE reads from 30 whole-genome sequencing samples of conifers and *Gnetum gnemon* to assemble reference *kChains* (putative repetitive elements) with the tBiClustering algorithm. Since 10% of raw data is a large enough sample to represent general distribution of pair-kmers in the total dataset, we expect that the assembly of the repetitive pair-kmers that were generated from 10% of raw data will produce majority of the highly repetitive fragments that are contained in the total dataset.

The relatively short *kChain* sequences were extended by Trinity software [35]. We used Trinity, a transcriptome assembler, because we expect to encounter individual disconnected graphs, rather than few large connected sequences, each representing the repetitive element.

### 2.6. Principal Component Analysis (PCA)

The PCA method [36] performs compression of multidimensional distribution of points in space of smaller dimension (typically two-dimensional space) with minimal distortion of inter-point distances. Thus, the PCA analysis was performed for 30 dimensional points (10 species in 3 replicates)—these are log-abundance profiles of known (RepBase and PIER) or newly detected RE elements (*kChains*).

REs or *kChains* can also be assigned a position in the PCA space. This position is determined after linear orthogonal transformation of the original space into space with PCs as coordinates, and then compressing the initial space to a space based on first two PCs as new coordinates of the space. The selected PCs cover highest fraction of the total data variability.

PCA analysis allows associating columns and rows of the analyzed abundance matrix through their positions on the PCA space: coordinates of columns are their loadings on the selected PC-components, and coordinates of rows are their coordinates as points in the linearly transformed and compressed space [36]. PCA analysis gave positions of species (columns of the analyzed table) and positions of repetitive elements (rows of the table). The species and repetitive elements that have the maximal abundances in these species are associated as they occupy the same region on the PCA plane.

### 2.7. Phylogenetic Analysis Based on Repeat Abundance

Phylogenetic analyses based on the abundance of known RepBase and PIER repeats and novel putative repeats (*kChains*) were created using the neighbor-joining method from R package APE [37].

## 3. Results

### 3.1. Abundance of Known RE in Conifers and Gnetum Species

#### 3.1.1. Clustering of Species on the PC1-PC2 Plane Agrees with the Taxonomy

**Figure 2:**
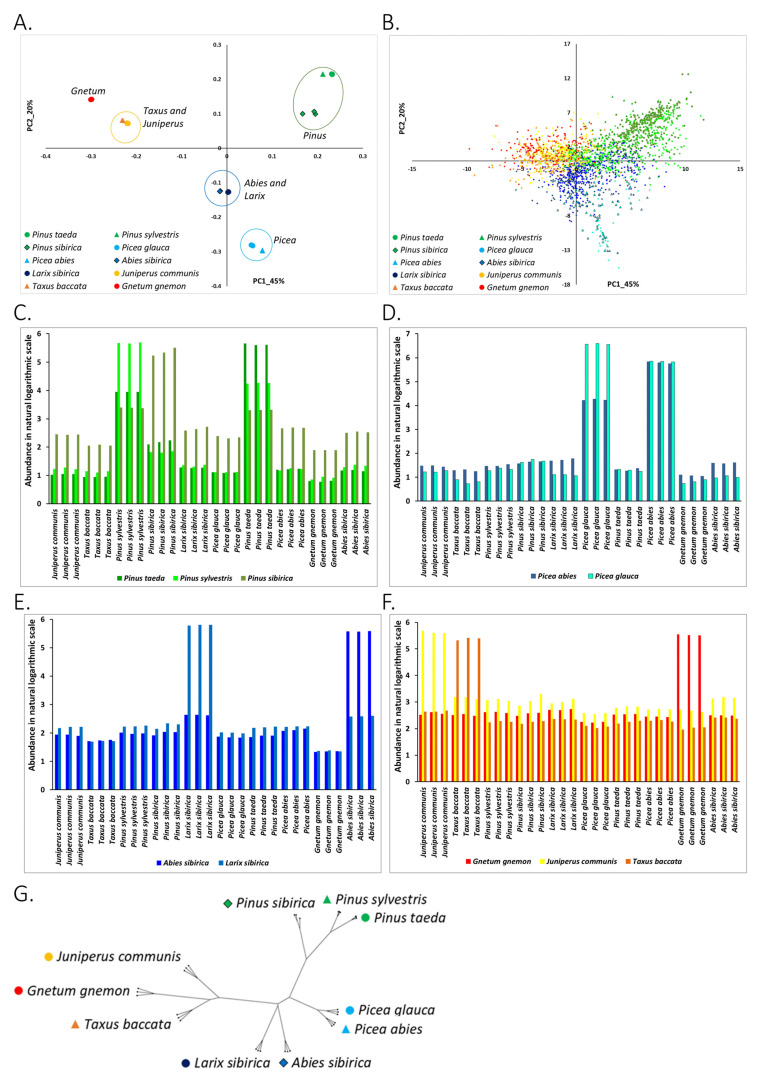
Known repetitive elements (RepBase and PIER) in conifers and *Gnetum gnemon* species. (**A**) PCA projection of samples based on log-abundances of known repeats from RepBase and PIER databases. Abundance for all repeats was calculated as per position average of counts of mapped reads. A repeat was filtered out from the table if its maximum abundance across samples was less than a threshold (150 reads). Points are samples, and colors indicate the plant species that the sample belongs to. (**B**) Projection of known RE on the PC1-PC2 plane. Dots on the graph represent repeats, and they are colored according to the species that this repeat is most represented in. For example, repeats colored in red have maximum abundance in samples of *Gnetum gnemon*. Positions of the repetitive elements on the PC plane show their association with plant species. (**C**) Abundance profiles of repeats that are most represented in *Pinus* species. Bars represent the average abundance of repeats most represented in *Pinus* species across all samples. Repeats with maximum abundance in *Pinus taeda* are colored in green, repeats with maximum abundance in *Pinus sylvestris* are colored in light green, and repeats with maximum abundance in *Pinus sibirica* are colored in olive green. Repeats that have maximum abundance in *Pinus* species are present in all other conifers. *Pinus taeda* and *Pinus sibirica* are interconnected by repeats: repeats with maximum abundance in *Pinus taeda* are also highly abundant in *Pinus sylvestris* samples. Additionally, repeats with maximum abundance in *Pinus sylvestris* are abundant in *Pinus taeda* samples. *Pinus sibirica* species is an outlier in genus *Pinus* and represents subgenus *Strobus*, unlike *Pinus taeda* and *Pinus sylvestris*, which belong to subgenus *Pinus*. (**D**) Abundance profiles of repeats most represented in *Picea* species. Bars represent the average abundance of repeats most represented in *Picea* species across all samples. Repeats with maximum abundance in *Picea abies* are colored in bright blue, repeats with maximum abundance in *Picea glauca* are colored in light blue. Association of *Picea* species by abundance of repeats: repeats with maximum abundance in *Picea glauca* are also highly abundant in *Picea abies*; however, repeats highly abundant in *Picea abies* species have much lower abundance in *Picea glauca*. (**E**) Abundance profiles of repeats that are most represented in *Abies* and *Larix* species. Bars represent the average abundance of repeats most represented in *Abies* and *Larix* across all samples. Repeats with maximum abundance in *Abies sibirica* are colored in dark blue, repeats with maximum abundance in *Larix sibirica* are colored in blue. *Abies* and *Larix* have species-specific repeats. (**F**) Abundance profiles of repeats most represented in *Gnetum gnemon, Juniperus communis,* and *Taxus baccata* species. Bars represent the average abundance of these repeats across all samples. Repeats with maximum abundance in *Gnetum gnemon* are colored in red, repeats with maximum abundance in *Larix sibirica* are colored in yellow, and repeats with maximum abundance in *Taxus baccata* are colored in orange. *Gnetum*, *Juniperus,* and *Taxus* have species-specific repeats. (**G**) Phylogenetic tree of studied species based on the abundance of known RE.

Aligning the genomic reads on the two databases of known repetitive elements, RepBase and PIER (see Section 2), we identified more than 7000 highly abundant (more than 150 reads per base pair at least in one sample) known RE (Table S2). Principle component analysis (PCA) revealed that the abundance of known RE is well associated with the species’ taxonomy (Figure 2A). The replicates of all species were tightly clustered into four groups on the PC1-PC2 plane that covered 65% of total data variability (Figure 2A). The first group contained all three *Pinus* species. The second group included the two *Picea* species. Based on the abundance of known repetitive elements, this *Picea* group is farther from *Pinus* than the combined *Larix-Abies* group. This finding does not match the known phylogeny based on chloroplast genomics, where *Pinus* and *Picea* species are closer to each other than *Pinus* is to *Larix* and *Abies* species [38]. The third group included the *Larix sibirica* and the *Abies sibirica* samples. Phylogenetically, *Larix sibirica* and *Abies sibirica* belong to closely related groups: *Laricoideae* and *Abitoideae*, respectively. Finally, the fourth group included the *Taxus* and *Juniperus* samples with *Gnetum gnemon* close by (Figure 2A). This group was separated from the three others, mirroring their taxonomic distance from other species (Figure S2 adapted from [39]).

#### 3.1.2. Association of Types of Known RE with the Studied Plants

PC analysis can be used to link the repetitive elements with the groups of plants based on the position of repeats and plant samples on the PC plane (Section 2). Projection of known RE on the PC1-PC2 plane yielded a link of known RE with plant species (Figure 2B). Abundance profiles of repeats across all samples are presented in Figure 2C–F. Bar graphs denote the average abundance of repeats most represented (most abundant) in some particular species (depending on the color of the bars) across all samples. For example, in Figure 2C, green bars represent the average abundance of repeats that have maximum abundance in *Pinus taeda* across all samples. In fact, these repeats, when projected on the PC1-PC2 plane, occupy the same position as samples of *Pinus taeda* on the PC1-PC2 plane.

We found that repeats from RepBase and PIER databases were present in all studied gymnosperm and *Gnetum* species. However, they were much more abundant in the species associated with their location on the PCA plane. Based on the abundance of RepBase and PIER repeats, the most linked groups of species were *Pinus*, mostly *Pinus taeda* and *Pinus sylvestris* (Figure 2C). Repeats with maximum abundance in *Pinus taeda* were highly abundant also in *Pinus sylvestris* and vice versa. *Pinus sibirica* appears to be an outlier in the *Pinus* group. RepBase and PIER repetitive elements associated with *Pinus sibirica* were highly abundant in two other *Pinus* species but not vice versa (Figure 2C). This may be anticipated as *Pinus sibirica* belongs to the *Strobus* subgenus and the other two *Pinus* species belong to the *Pinus* subgenus.

Similarly, the *Picea* species (*Picea glauca* and *Picea abies*) were linked by the abundant RepBase and PIER repeats. Namely, highly abundant repeats in *Picea glauca* were also very abundant in *Picea abies*, and vice versa (Figure 2D).

Other biological groups of species were not internally linked by the most organism-specific RepBase and PIER repeats (Figure 2E,F). *Larix sibirica, Abies sibirica, Taxus, Juniperus,* and *Gnetum gnemon* all had species-specific abundant known repeats.

Repeats from RepBase and PIER databases can be assigned to repeat families and super-families. This annotation of known repeats may further clarify whether different conifer species accumulate repetitive elements from different super-families. Indeed, we were able to identify highly abundant repeats from different super-families including terminal inverted repeats (TIRs), mutator-like transposable elements (MULEs), long interspersed nuclear elements (LINEs), Helitrons, LTR/Gypsi, LTR/Copia, LTR/ERV1, hAT transposons, CACTA transposons, and others. However, we did not find an association of specific repeat families with plant species. Almost all families of repeats were distributed across the PC1-PC2 plane (Figure S1). Thus, our analysis revealed that repeats from most families were present in all species of studied plants with comparable abundance (Figure S1). Indeed, while specific repeats were species-specific, whole families of repeats were not.

#### 3.1.3. Phylogenetic Analysis of Conifer and *Gnetum* Species Based on the Genomic Abundance of Known RE

Next, we constructed a phylogenetic tree based on the abundances of known RE in genomes of the studied plants. Phylogeny based on the abundance of repetitive sequences in genomes of plants and other organisms has been suggested to be useful as an additional signal of organism evolution together with more classical sequence-based phylogenetic inferences [40]. We found that the phylogenetic tree inferred from the abundance of known RE in the genomes of conifer and *Gnetum* species (Figure 2G) matches fairly well with the known phylogeny based on sequences of all chloroplast coding genes [38], and on sequences of chloroplast *rbcL* and *matK* genes [41], summarized in a simplified tree in Figure S2. However, our phylogenetic analysis based on abundance of known RE indicated that *Taxus* and *Gnetum gnemon* had the most recent common ancestor (MRCA). This is not supported by previous studies based on sequence similarities of single-copy genes [42] and on chloroplast genome sequences [38,41] which show that *Taxus* and *Junniperus* species are the most closely related. Additionally, the speciation of *Abies* and *Larix* did not match the known phylogeny. The *Abies-Larix* group in our analysis formed a clade. However, according to previous studies, the speciation of *Abies* from *Pinus-Picea* had happened before the speciation of *Larix* from *Pinus-Picea* [38,41]. Speciation of *Pinus*-*Picea* group was in good match with the known phylogeny. One *Pinus sibirica* sample that was not preprocessed to provide an internal control (see Section 2), separated from other *Pinus sibirica* samples earlier on, suggesting the importance of preprocessing raw genome sequencing reads from adapters and other technical artifacts before alignment on reference sequences.

The taxonomic and the phylogenetic relations between conifer and *Gnetum* species based on the analysis of the abundance of known RE in the genomes of these plants can suffer from several biases, especially biases related to the loss of information about the true diversity of repetitive elements in the genome. Thus, as the next step of our investigation, we studied the “dark repeatome” in the genomes of the ten chosen conifer and *Gnetum* species more closely.

### 3.2. “Dark Repeatome”: Its Abundance in Genomes of Studied Plants

To investigate the full spectrum of “dark repeatome” of the ten studied plants, we utilized the tBiClustering algorithm [34] for the detection of highly repetitive sequences in the genomic reads (*kChains*) of the studied plants (see Section 2). After identifying the highly repetitive sequences, we aligned all reads previously unmapped on the RepBase and PIER databases onto these constructed *kChains* and generated an abundance matrix of these putative repetitive elements. We next transformed the matrix to natural logarithmic scale, quantile normalized it, and chose only highly abundant (>665 at least in one sample) *kChains* for further analyses (see Section 2).

Highly repetitive whole-genome sequencing reads can be a result of several biological and technical mechanisms. Namely, these repetitive sequences may be short pieces of repeats present in the plant genome (true repetitive elements). In addition, highly repetitive reads can originate from chloroplast or mitochondrial genomes. These sequences can also result from technical artifacts such as primers or adapters used for the sequencing but which were not included in the Trimmomatic adapter list and thus were not detected and removed by Trimmomatic.

To identify the possible confounding sequences assembled as *kChains,* we annotated all *kChains* by aligning them to publicly available cpDNA assemblies (Table S3). *kChains* that were not aligned to cpDNA assemblies were further annotated by aligning them to the ‘nr/nt’ database [43] with BLAST [44]. Most of the successfully annotated *kChains* unexpectedly aligned to the *Cyprinus carpio* genome and were most abundant in the *Juniperus communis* samples (Figures S2 and S3, Table S4). To reduce the confounding factor of the contaminations of putative repetitive elements, we removed *kChains* that aligned onto cpDNA, mtDNA, and other confounding sequences from ‘nr/nt’ database from further analysis. In this way, the final *kChain* abundance matrix used for downstream analyses included only nuclear highly abundant DNA repetitive elements (Table S5).

We identified 9928 highly abundant (see Section 2) *kChains*, 24% of which (2413) were unique to one species. Another 127 *kChains* were present in all species. The other *kChains* were present in at least two different species (Table S5).

#### 3.2.1. Clustering of Species on the PC1-PC2 Plane and Association of Species with Nuclear DNA *kChains*

**Figure 3:**
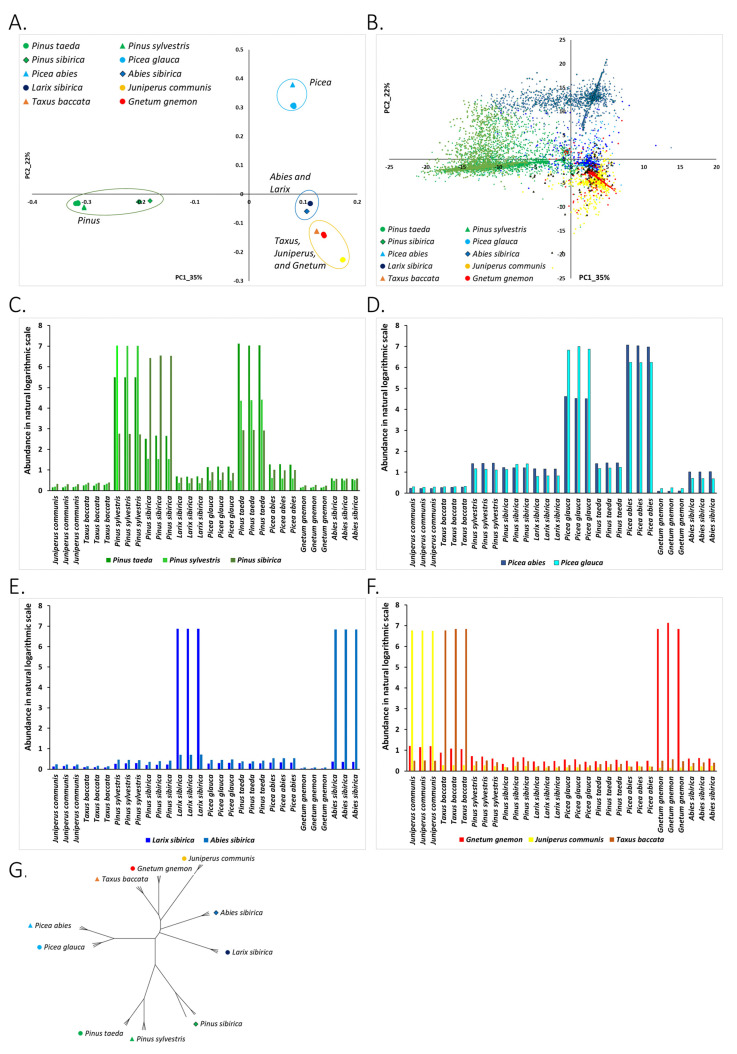
Putative repetitive elements (*kChains*) in conifers and *Gnetum* species. (**A**) PCA projection of samples based on log-abundances of newly detected putative RE (*kChains*). Abundance for *kChains* was calculated as per position average of counts of mapped reads. A *kChain* was filtered out from the table, if its maximum abundance across samples was less than a threshold (665 reads). Points are samples, and colors indicate the plant species that the sample belongs to. (**B**) Projection of *kChains* on the PC1-PC2 plane. Dots on the graph represent *kChains*, and they are colored according to the species that this repeat is most represented in. For example, *kChains* colored in red have maximum abundance in samples of *Gnetum gnemon*. Positions of the *kChains* on the PC plane show their association with plant species. (**C**) Abundance profiles of *kChains* that are most represented in *Pinus* species. Bars represent the average abundance of *kChains* most represented in *Pinus* species across all samples. *kChains* with maximum abundance in *Pinus taeda* are colored in green, *kChains* with maximum abundance in *Pinus sylvestris* are colored in light green, *kChains* with maximum abundance in *Pinus sibirica* are colored in olive green. *kChains* that have maximum abundance in *Pinus* species are present in all other conifers. *Pinus taeda* and *Pinus sibirica* are interconnected by *kChains*. *kChains* with maximum abundance in *Pinus taeda* are also highly abundant in *Pinus sylvestris* samples. Additionally, *kChains* with maximum abundance in *Pinus sylvestris* are abundant in *Pinus taeda* samples. *Pinus sibirica* species is an outlier in genus *Pinus* according to the abundance of *kChains*. Indeed, *Pinus sibirica* belongs to subgenus *Strobus*, unlike *Pinus taeda* and *Pinus sylvestris*, which belong to subgenus *Pinus*. (**D**) Abundance profiles of *kChains* most represented in *Picea* species. Bars represent the average abundance of *kChains* most represented in *Picea* across all samples. *kChains* with maximum abundance in *Picea abies* are colored in bright blue, *kChains* with maximum abundance in *Picea glauca* are colored in light blue. *Picea glauca* and *Picea abies* were strongly linked by *kChains*, but not symmetrically; *kChains* of *Picea glauca* were of higher abundance in *Picea abies* than the other way around. (**E**) Abundance profiles of repeats that are most represented in *Abies* and *Larix* species. Bars represent the average abundance of repeats most represented in *Abies* and *Larix* species across all samples. Repeats with maximum abundance in *Abies sibirica* are colored in dark blue, repeats with maximum abundance in *Larix sibirica* are colored in blue. *Larix sibirica* and *Abies sibirica* species were found to have high-specificity *kChains*, *kChains* most abundant in *Abies sibirica* samples were not abundant in *Larix sibirica*, and vice versa. (**F**) Abundance profiles of *kChains* most represented in *Gnetum gnemon, Juniperus communis,* and *Taxus baccata* species. Bars represent the average abundance of these *kChains* across all samples. *kChains* with maximum abundance in *Gnetum gnemon* are colored in red, *kChains* with maximum abundance in *Larix sibirica* are colored in yellow, and *kChains* with maximum abundance in *Taxus baccata* are colored in orange. *Gnetum*, *Juniperus,* and *Taxus* have species-specific *kChains*. (**G**) Phylogenetic tree of studied species based on the abundance of *kChains*.

PC analysis based on the abundance of nuclear DNA *kChains* gave taxonomically meaningful separation of species on the PC1-PC2 plane (Figure 3A). *Pinus* species were grouped together. Additionally, the two *Picea* species formed a tight group on the PC1-PC2 plane. *Abies* and *Larix* species were also grouped. Similar to our previous analysis of known RE, we found an association between putative new nuclear DNA repeats (*Kchains*) and the conifer species on the PCA plane (Figure 3B). Namely, on the PC1-PC2 plane, *kChains* with maximum abundance in a species were closely positioned to the samples of this species (Figure 3A,B). Interestingly, one can note that clusters of *Kchains* were much more tightly linked to groups of conifer species than the RepBase and PIER repeats. For example, *Picea*-specific *kChains*, *kChains* with maximum abundance in *Picea abies* and *Picea glauca* species, were highly abundant only in *Picea* with low abundance in other species (Figure 3D). This specificity of *kChains* to the species of plants may be a result of high specificity of short repetitive sequences. *kChains* were short (30–200 bp) repeats which may include truncated RE, short satellite DNA, and other short and highly repetitive sequences.

#### 3.2.2. Phylogenetic Analysis of Conifer and *Gnetum* Species Based on The Genomic Abundance of Putative Repetitive Elements (*kChains*)

The phylogenetic tree inferred from the abundance of putative repetitive elements (*kChains*) resembled the tree constructed from the known RE (Figure 2G) with greater specificity of species that reflect the known conifer phylogenetics. Early separation of *Abies* from *Larix* samples, in contrast to *Larix-Abies* clade formation when the abundance of known (RepBase and PIER) repeats were used, matches the phylogeny described before [38,41]. *Abies* and *Larix* speciation, based on *kChain* abundance, was followed by the two *Picea* species, and later by *Pinus* species. *Pinus sibirica* differentiated first, which matches the known *Pinus* phylogeny. This greater sensitivity of phylogenetic analysis based on the abundance of *kChains* aligns with the greater specificity of *kChains* to plant species reflected by PCA separation in Figure 3B. Our phylogenetic analysis based on the abundance of putative repetitive elements (*kChains*) in genomes of conifer and *Gnetum* species revealed that the *Pinus-Picea* clade is monophyletic. In addition, we found that the *Pinus-Picea-Larix* clade is also monophyletic. The *Pinus sibirica* speciation from species is evident and has occurred earlier according to *kChain* abundance (Figure 3G) compared to RepBase and PIER repeat abundances (Figure 2G).

Our phylogenetic analysis based on the abundance of short *kChains* may help clarify the interspecies relationship. It is more sensitive to genomic variations than analysis based on already well identified and classified known repeats from RepBase and PIER databases.

#### 3.2.3. Analysis of *kChains* Associated with Chloroplast Genomes

As mentioned above, we removed the *kChains* aligned to cpDNA from our previously described analyses. However, cpDNA contains many repetitive elements; thus, the abundance of cpDNA annotated *kChains* may be used similarly to nuclear repeats [45]. We hypothesized that the *kChains* aligning to cpDNA might reflect the taxonomy of the studied plants.

**Figure 4:**
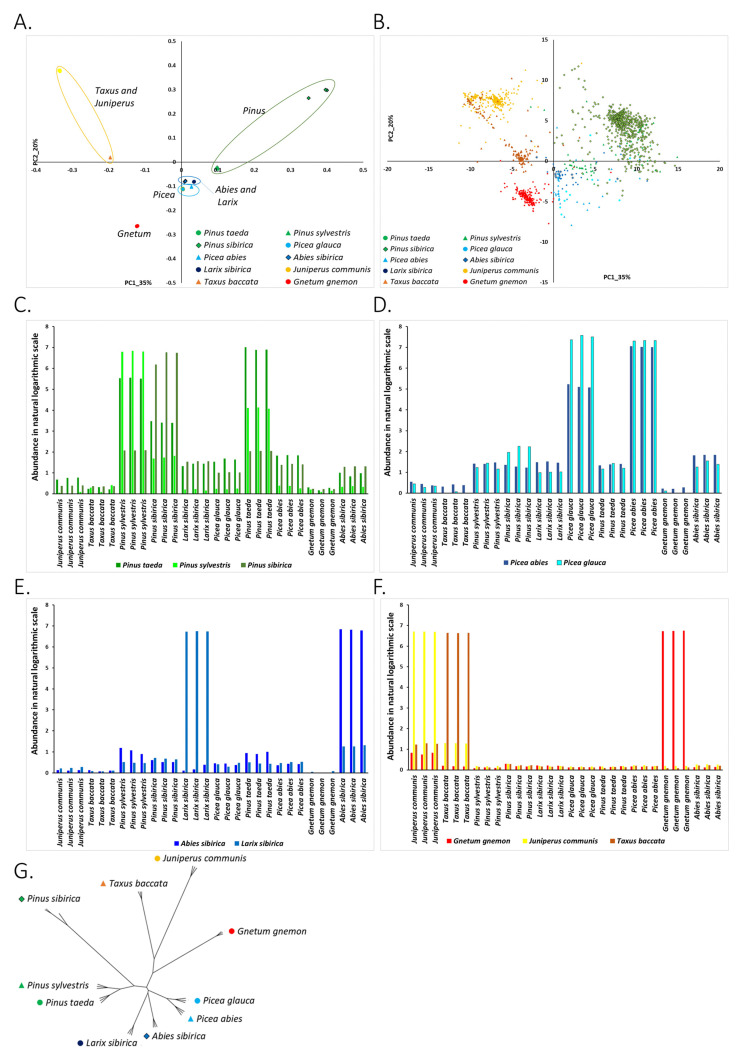
Putative repetitive elements (*kChains*) aligning to cpDNA assemblies. (**A**) PCA projection of samples based on log-abundances of cpDNA-*kChains*. Points are samples, and colors indicate the plant species that the sample belongs to. (**B**) Projection of cpDNA-*kChains* on the PC1-PC2 plane. Dots on the graph represent cpDNA-*kChains*, and they are colored according to their maximum abundance. For example, *kChains* colored in red have maximum abundance in samples of *Gnetum gnemon*. Positions of the *kChains* on the PC plane show their association with plant species. (**C**) Abundance profiles of cpDNA-*kChains* that have maximum abundance in *Pinus* species. cpDNA-*kChains* with maximum abundance in *Pinus taeda* are colored in green, cpDNA-*kChains* with maximum abundance in *Pinus sylvestris* are colored in light green, and cpDNA-*kChains* with maximum abundance in *Pinus sibirica* are colored in olive green. cpDNA-*kChains* that have maximum abundance in *Pinus* species are present in all other conifers. *Pinus taeda* and *Pinus sibirica* are linked by high abundance cpDNA-*kChains*: cpDNA-*kChains* with maximum abundance in *Pinus taeda* also very abundant in *Pinus sylvestris* samples. Additionally, cpDNA-*kChains* with maximum abundance in *Pinus sylvestris* are highly abundant in *Pinus taeda* samples. *Pinus sibirica* species is an outlier in *Pinus* genera. (**D**) Abundance profiles of cpDNA-*kChains* that have maximum abundance in *Picea* species. cpDNA-*kChains* with maximum abundance in *Picea abies* are colored in bright blue, cpDNA-*kChains* with maximum abundance in *Picea glauca* are colored in light blue. Association of *Picea* species by abundance of cpDNA-*kChains*: cpDNA-*kChains* with maximum abundance in *Picea glauca* are also highly abundant in *Picea abies*, however cpDNA-*kChains* with maximum abundance in *Picea abies* have much lower abundance in *Picea glauca*. (**E**) Abundance profiles of cpDNA-*kChains* that have maximum abundance in *Abies* and *Larix* species. cpDNA-*kChains* with maximum abundance in *Abies sibirica* are colored in dark blue, cpDNA-*kChains* with maximum abundance in *Larix sibirica* are colored in blue. *Abies* and *Larix* have species-specific *kChains*. (**F**) Abundance profiles of cpDNA-*kChains* that have maximum abundance in *Gnetum gnemon, Juniperus communis,* and *Taxus baccata* species. cpDNA-*kChains* with maximum abundance in *Gnetum gnemon* are colored in red, cpDNA-*kChains* with maximum abundance in *Larix sibirica* are colored in yellow. cpDNA-*kChains* with maximum abundance in *Taxus baccata* are colored in orange. *Gnetum*, *Juniperus,* and *Taxus* have species-specific *kChains*. (**G**) Phylogenetic tree of studied species based on the abundance of chloroplast putative RE (cpDNA-*kChains*).

The separation of plant species on the PCA plane based on the abundance of chloroplast-associated *kChains* revealed that *Juniperus-Taxus-Gnetum* species, which were grouped based on the abundance of known and putative nuclear DNA REs, were distant based on the abundance of cpDNA *kChains* (Figure 4A). *Picea* species were somewhat similar to *Abies* and *Larix* species based on cpDNA *kChains* abundances; however, based on genomic *kChain* abundances, *Picea* species were very distant. *Abies* and *Larix* were grouped together regardless of repeat abundance source (*kChains*, cpDNA *kChains*, or known REs). *Pinus sibirica* was also distant from two other *Pinus* species and a representative of another subgenus. This was more pronounced in the cpDNA analysis (Figure 4A,G).

Association of chloroplast *kChains* with the plant species by their location on the PCA plane revealed that chloroplast-associated *kChains* are taxa-specific (Figure 4B–F). It may be that the abundance of found *kChains* reflects the similarities between chloroplast genomes of the same taxa and the differences between chloroplast genomes of different taxa [45].

The phylogenetic relationship of *Taxus-Juniperus-Gnetum* species based on the abundance of cpDNA *kChains* (Figure 4G) was similar to previously known [41]. However, the phylogenetic tree based on the abundance of cpDNA *kChains* pointed to peculiar speciation of *Picea-Abies-Larix* group.

## 4. Discussion

Repetitive elements (REs) are highly abundant in plant genomes and are the primary sources of intra- and inter-species genetic variations [21]. Here, we investigated the inter-species relationships based on the abundance of repetitive genomic elements in conifers and *Gnetum gnemon* genera. We showed that the abundance of genomic RE reflects the evolution of plant genomes and carries phylogenetic signals which may be used in addition to more classical approach of sequence similarities.

The approach we utilized in this study may be used for detecting the abundance of known and newly assembled RE from whole genome sequencing reads. Raw genomic reads were first cleaned from adaptors and other technical sequences. Next, cleaned reads were aligned to known repeats from RepBase [32] and PIER [7] databases. The reads that were not successfully aligned were analyzed using a sensitive unsupervised bi-clustering (tBiClustering) procedure and assembled into putative short repetitive elements (*kChains*).

It should be noted that we counted NGS reads aligned to repeats (repeat-reads). These counts were normalized by taking 10 Gb of raw reads in each samples. By this way, actually, we normalized numbers of estimated repeat-integrations by genome lengths assuming their uniform coverage across genome and that the number of repeat-reads is proportional to a number of repeat-integrations in the genome. In a larger genome the level of coverage per 10 Gb of raw reads is lower than in a smaller one. The same number of integrations in a larger genome will produce lower number of repeat-reads. If in a larger genome, the number of repeat-reads is the same or higher than in a smaller one, it means that a larger genome has more integration sites. Therefore, by finding repeat-reads in the same amount of raw reads in each genome we indirectly normalized numbers of repeat-integrations by genome lengths via estimating numbers of integration sites in the same length-units of all genomes.

The distribution and patterns of abundance profiles of the putative repetitive elements (*kChains*) across species (Figure 3) were compared to the distribution and abundance patterns of annotated elements from RepBase and PIER databases (Figure 2). The PCA grouping of species according to *kChains* and known repetitive elements abundance profiles was biologically meaningful and in consensus with the conifer taxonomy. *Pinus* and *Picea* species matched the known confer taxonomy, the grouping of *Larix sibirica* and *Abies sibirica* species was in good agreement with their known phylogeny, and clustering of *Cypress* (*Juniperus communis* and *Taxus baccata speacies*) and *Gnetum* species was also in a reasonable correspondence with their phylogeny [38,41].

The highly abundant known and putative REs that were shared between species suggest that similar genome evolutionary forces shaped those species (Figure 2C–F and Figure 3C–F). The group of pines was tightly linked by the *Pinus* taxon-specific known repeats (Figure 2C) and putative repetitive elements (*kChains*) (Figure 3C). However, *Pinus sibirica* separated from the other two *Pinus* species, and highly abundant known and putative repetitive elements in *Pinus sibirica* were not as plentiful in the other two *Pinus* species (Figure 2C and Figure 3C). Indeed, *Pinus sibirica* belongs to the subgenus *Strobus*, while the other two pine species belong to a subgenus *Pinus*.

*Picea glauca* and *Picea abies* were strongly linked by RE, but not symmetrically: both known and putative repeats of *Picea glauca* were of high abundance in *Picea abies*; however, *Picea abies* abundant repeats were not very abundant in *Picea glauca* (Figure 2D and Figure 3D).

*Larix sibirica* and *Abies sibirica* had very individual species-specific repertoires of highly abundant known and putative REs (Figure 2E and Figure 3E).

*Cypress* and *Gnetum* species were also enriched by highly abundant species-specific REs (Figure 2F and Figure 3F), matching their known phylogenetic separation. These results again indicate that the abundance of repetitive elements in the genomes of plants reflects the specific evolutionary forces acting on them.

Several species (mainly *Pinus sibirica* and *Gnetum gnemon*) were highly enriched with newly determined repeats (*kChains*) associated with chloroplast. This may be expected as cpDNA is highly abundant in plant cells and is represented by several hundred or sometimes even thousand copies (organelles) per cell, unlike a single nuclear genome copy per cell [46]. Interestingly, the clustering of plant species on the PC1-PC2 plane based on the abundance of cpDNA *kChains* was similar to the clusters formed based on the abundance of known and putative nuclear genome repeats (Figure 4A). The association of cpDNA *kChains* with plant species (Figure 4C–F) was also in a good match with the similar association of repeats with plant species (Figure 2C–F and Figure 3C–F).

As a by-product of the analysis of repetitive elements in conifer plants, we revealed that many of the *kChains* aligned well to *Cyprinus carpio* genomic sequences. These *kChains* were enriched mostly in the *Juniperus communis* samples (Figure S3). The *Cyprinus carpio* assembled genome is known to be contaminated by the Illumina adaptors [47], and we hypothesize that the identified *kChains* are these adaptors which were not cleaned by the Trimmomatic tool [31] because they were not part of the default adapter list. Indeed, often, the exact adaptor sequences that were used are not known to the researcher, especially if the re-analysis of data is performed. Therefore, we propose that the tBiClustering algorithm may be used as an additional tool for detecting and cleaning highly repetitive artifact sequences from NGS reads.

## 5. Conclusions

Raw genomic sequencing data contain rich information about the “repeatome”. This information is frequently unavailable in already assembled genomes because genomic areas of repeats are often masked in genome assembly and annotation. Many of the plant genomes are extremely abundant in repetitive DNA. This vast “repeatome” may play an essential role in regulating plant evolution and adaptation. In a comparative genomics study of repetitive elements of different plant genera, the application of the tBiClustering algorithm to the massive pool of raw sequence data allowed us to efficiently detect repetitive elements and their abundance profiles across different species.

## Data Availability

All analyzed raw genomic data are available from NCBI database. Accession numbers are presented in Table S1.

## Supplementary Materials

The following are available online at https://www.mdpi.com/article/10.3390/life11111234/s1: Figure S1: Abundance of families of known repeats across plant species, Figure S2: A simplified phylogeny of studied genera adapted from Uddenberg et al. 2015 [39], Figure S3: PC1-PC2 distribution of *kChains* annotated as *Cyprinus carpio* genomic sequences, Table S1: Species taxonomy and links to the sequencing data used in the study, Table S2: Abundance matrix of RepBase and PIER repeats across 30 samples of conifers, Table S3: Abundance matrix of *cpDNA-kChains* across 30 samples of conifers, Table S4: Abundance matrix of *kChains* aligned to *Cyprino carpio* genomic sequences across samples of all plants, Table S5: Abundance matrix of nuclear genomic *kChains* across 30 samples of conifers.
