# Supplementary material for: Comparative Genomics Analysis of Repetitive Elements in Ten Gymnosperm Species: “Dark Repeatome” and Its Abundance in Conifer and Gnetum Species
Source: Life (Basel). 2021 Nov 15;11(11):1234. doi: 10.3390/life11111234 (PMC8620675; doi:10.3390/life11111234)
Supplement: Supplementary file 1 [file life-11-01234-s001.zip › Figure_S1_Abundance of families of known repeats across plant species.pdf]

A.

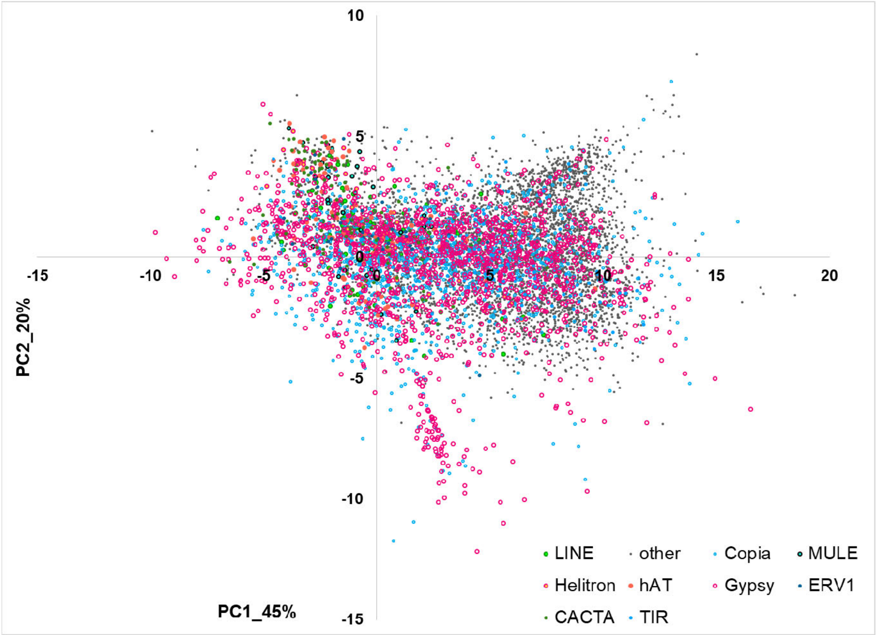

PC2_20%
PC1_45%
LINE
other
Copia
MULE
Helitron
hAT
Gypsy
ERV1
CACTA
TIR

B.

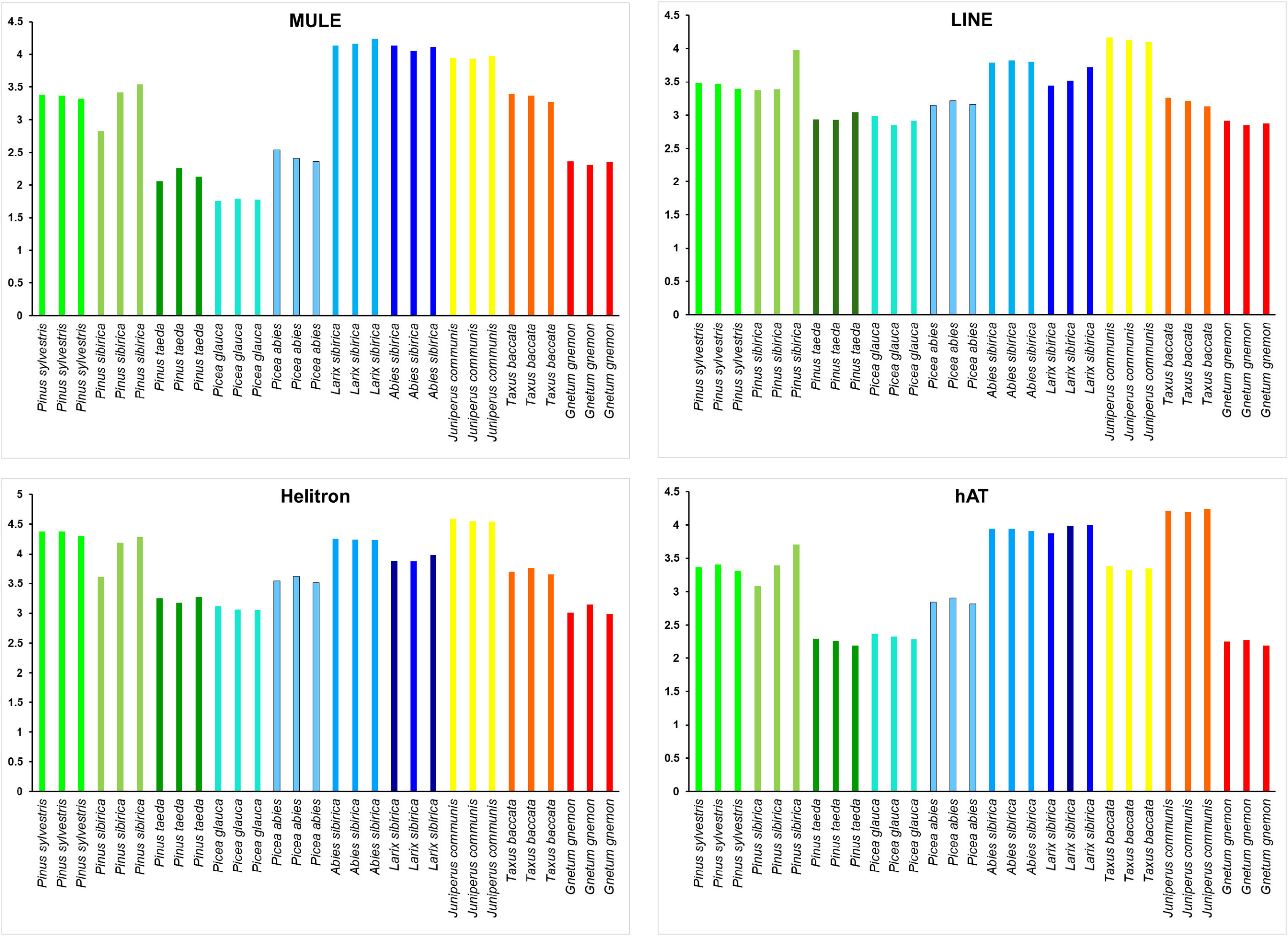

MULE
LINE
Helitron
hAT
Pinus sylvestris
Pinus sibirica
Pinus taeda
Picea glauca
Picea abies
Larix sibirica
Abies sibirica
Juniperus communis
Taxus baccata
Gnetum gnemon

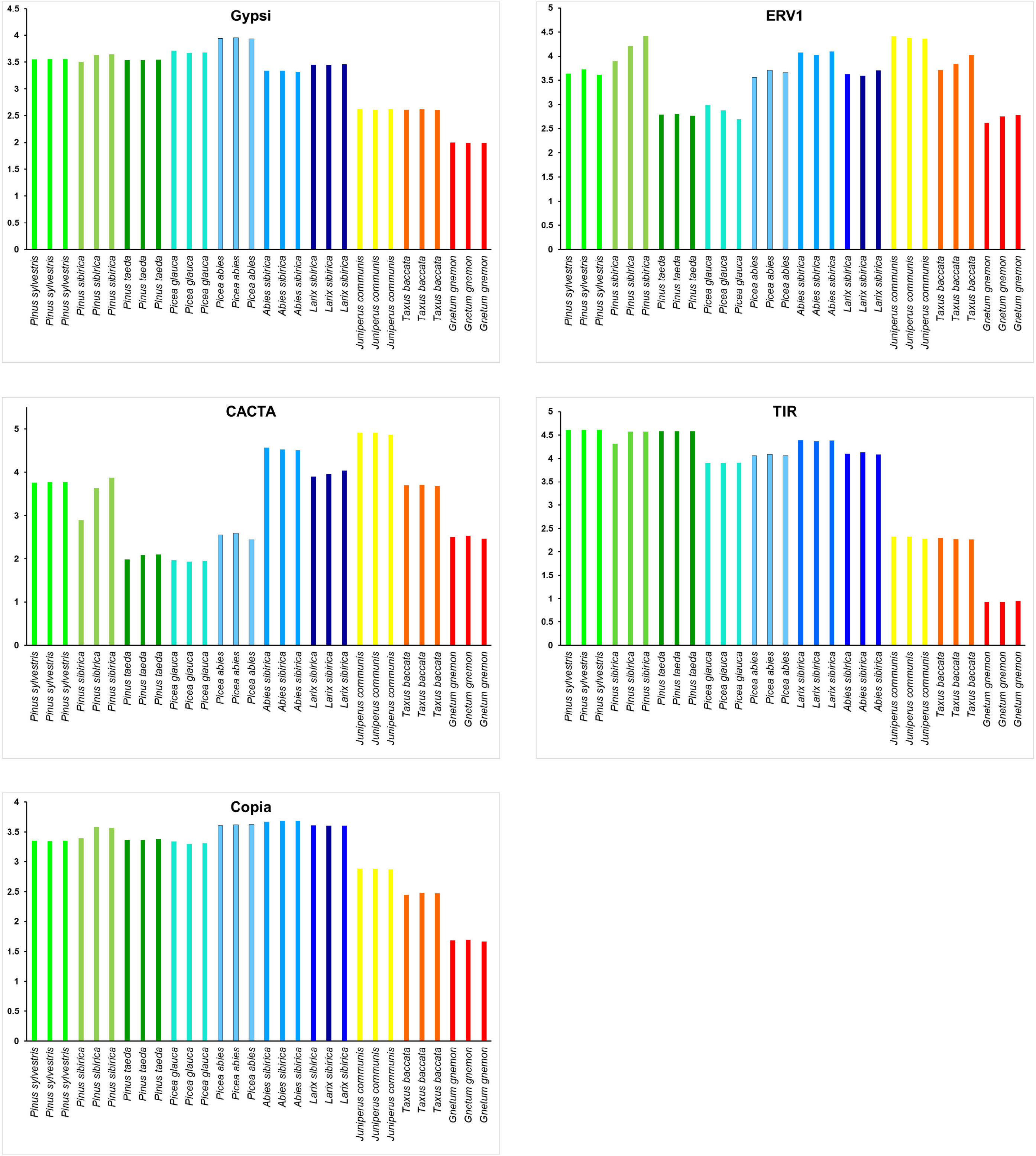

Figure S1. Abundance of families of known repeats across plant species

A. Projection of RepBase and PIER repeats on the PC1-PC2 plane. Dots on the graph represent repeats. Different colors denote repeats from different families.

B. Average abundance levels of repeats from different families across 30 plant species
