# Supplementary material for: Comparative Genomics Analysis of Repetitive Elements in Ten Gymnosperm Species: “Dark Repeatome” and Its Abundance in Conifer and Gnetum Species
Source: Life (Basel). 2021 Nov 15;11(11):1234. doi: 10.3390/life11111234 (PMC8620675; doi:10.3390/life11111234)
Supplement: Supplementary file 1 [file life-11-01234-s001.zip › Figure_S3_PC1-PC2 distribution of kChains annotated as Ciprinus carpio genomic sequences.pdf]

A.

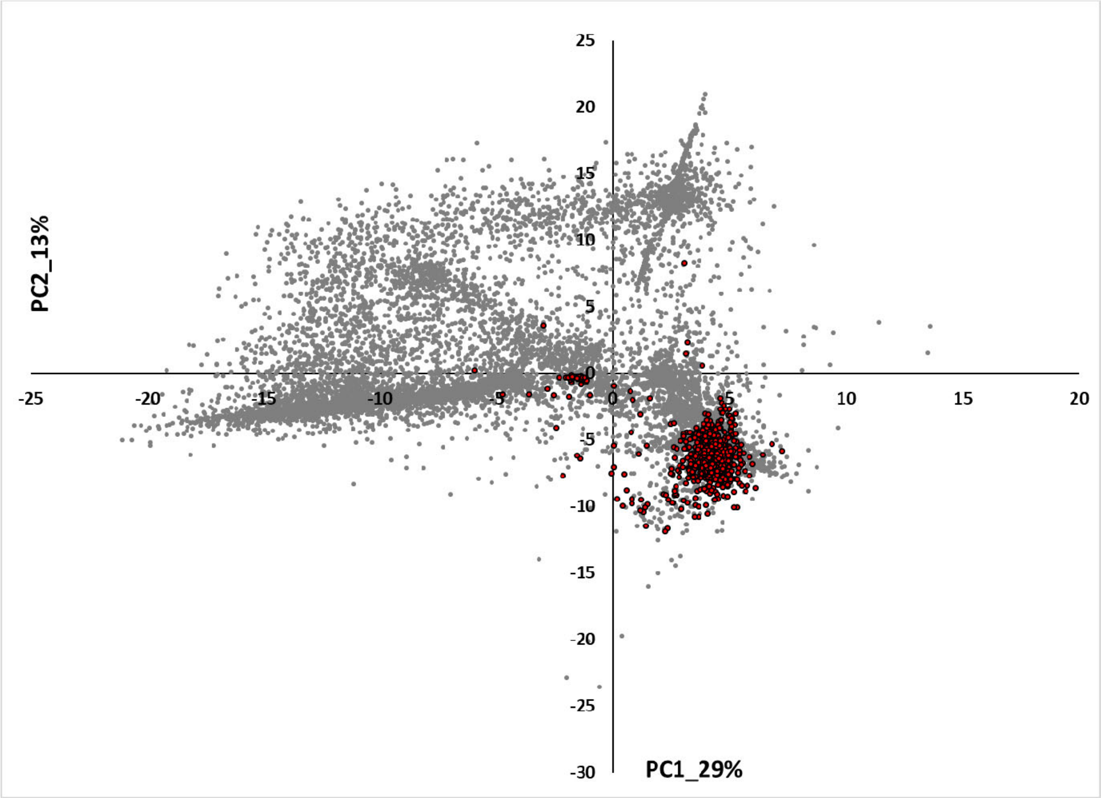

B.

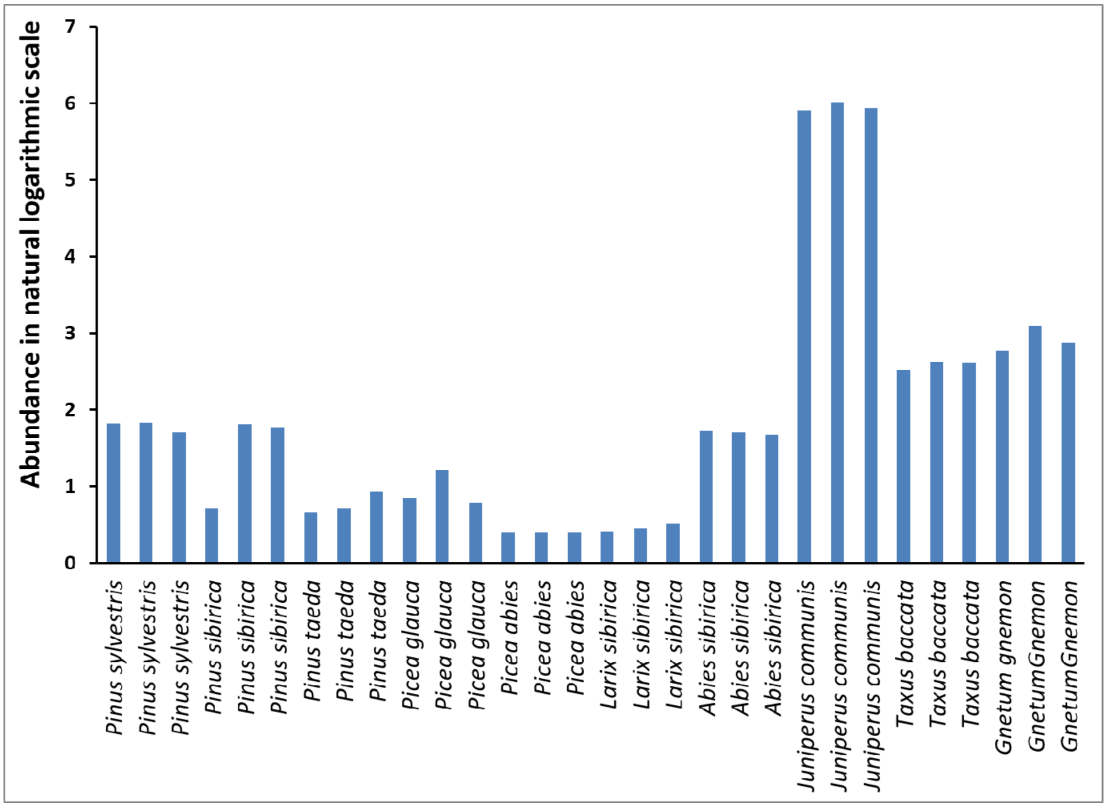

Figure S3. PC1-PC2 distribution of kChains annotated as Ciprinus carpio genomic sequences

A. Projection of *kChains* on the PC1-PC2 plane. Dots on the graph represent *kChains.* Dots colored red denote *kChains* annotated as *Cyprinus carpio* genome elements.

B. Average abundance levels of kChains annotated as *Cyprinus carpio.*
