# Supplementary figures and images for: Comparative Genomics Analysis of Repetitive Elements in Ten Gymnosperm Species: “Dark Repeatome” and Its Abundance in Conifer and Gnetum Species
Source: Life (Basel). 2021 Nov 15;11(11):1234. doi: 10.3390/life11111234 (PMC8620675; doi:10.3390/life11111234)

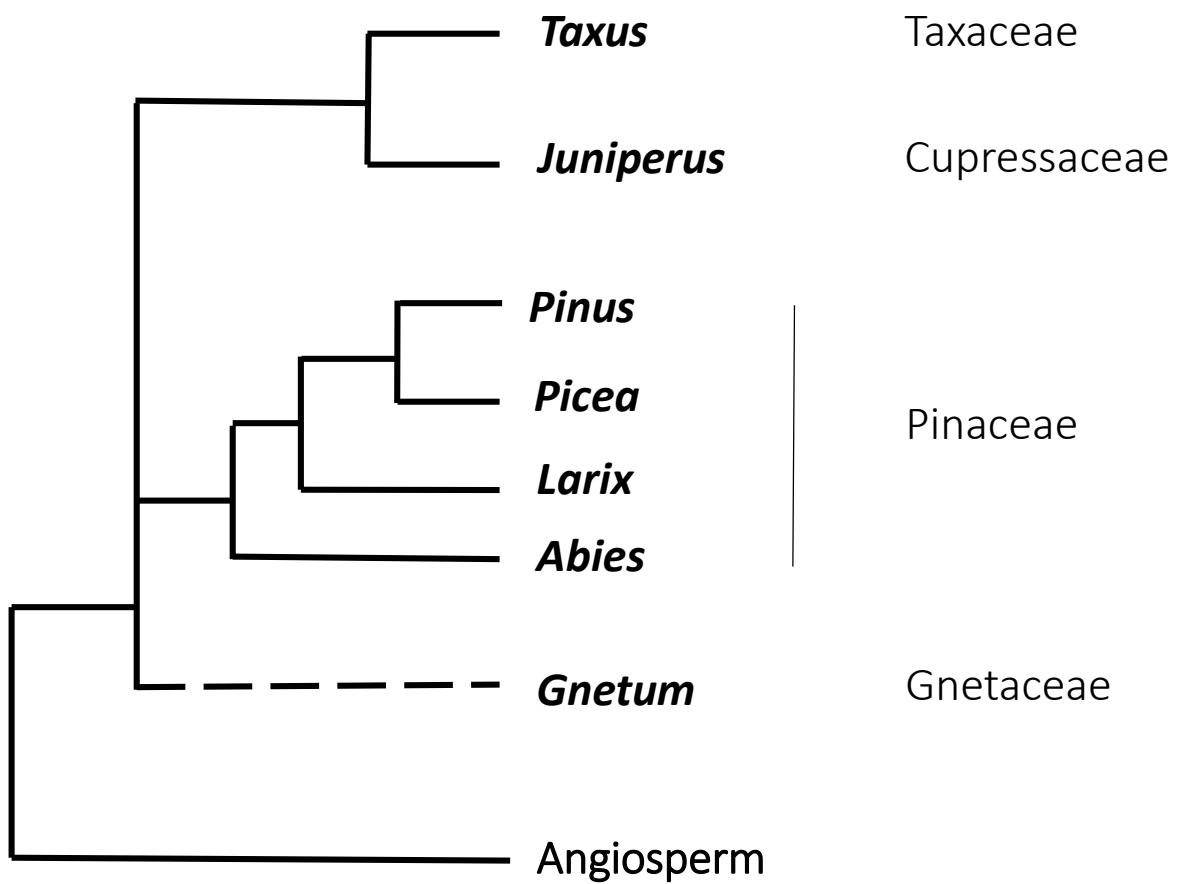

**FigureS2.** A simplified depiction of studied plant phylogeny adapted from Uddenberg et al, 2015 [39].

Supplement: Supplementary file 1 [file life-11-01234-s001.zip › Figure_S2_SimplifiedKnownPhylogeny.pdf]
